# Clarifying Boundaries of Binge Eating Disorder and Psychiatric Comorbidity: A Latent Structure Analysis

**DOI:** 10.1016/j.brat.2010.12.003

**Published:** 2010-12-21

**Authors:** Anja Hilbert, Denise E. Wilfley, Faith-Anne Dohm, Kathleen M. Pike, Christopher G. Fairburn, Ruth H. Striegel-Moore

**Affiliations:** a Department of Clinical Psychology and Psychotherapy, University of Fribourg, Rue P.-A. de Faucigny 2, 1700 Fribourg, Switzerland; b Department of Psychiatry, Unit 98, Columbia University, 1051 Riverside Drive, 10032 New York, New York, USA; c Department of Psychiatry, Washington University in St. Louis, 660 South Euclid, Campus Box 8134, 63110 St. Louis, Missouri, USW; d Department of Psychiatry, Warneford Hospital, Oxford University, OX3 7JX Oxford, UK; e Graduate School of Education & Allied Professions, Fairfield University, 1073 North Benson Road, 06824 Connecticut, Connecticut, USA; f Department of Psychology, Montana State University, PO Box 173440, 59717-3440 Bozeman, Montana, USA

**Keywords:** classification, psychiatric taxonomies, comorbidity, binge eating, eating disorders

## Abstract

Binge eating disorder (BED) presents with substantial psychiatric comorbidity. This latent structure analysis sought to delineate boundaries of BED given its comorbidity with affective and anxiety disorders. A population-based sample of 151 women with BED, 102 women with affective or anxiety disorders, and 259 women without psychiatric disorders was assessed with clinical interviews and self-report questionnaires. Taxometric analyses were conducted using DSM-IV criteria of BED and of affective and anxiety disorders. The results showed a taxonic structure of BED and of affective and anxiety disorders. Both taxa co-occurred at an above-chance level, but also presented independently with twice-as-large probabilities. Within the BED taxon, diagnostic co-occurrence indicated greater general psychopathology, lower social adaptation, and greater premorbid exposure to parental mood and substance disorder, but not greater eating disorder psychopathology. Eating disorder psychopathology discriminated individuals in the BED taxon from individuals in the affective and anxiety disorders taxon. Diagnostic criteria of BED were more indicative of the BED taxon than were criteria of affective and anxiety disorders. The results show that at the latent level, BED was co-occurring with, yet distinct from, affective and anxiety disorders and was not characterized by an underlying affective or anxiety disorder.

## Introduction

Classification of eating disorders is an enduring focus of debate. It relates to our understanding of the nature of pathology and its boundaries with other disorders, especially the preliminarily defined binge eating disorder (BED). Given BED’s substantial comorbidity with affective and anxiety disorders (Grilo, White, & Masheb, 2009; Javaras, Pope, Lalonde et al., 2008; Wilfley, Friedman, Dounchis et al., 2000), the current study sought to elucidate at the latent level whether BED represents an associated feature of affective and anxiety disorders, or a separate mental disorder.

Included in the Diagnostic and Statistical Manual of Mental Disorders fourth edition (DSM-IV) as a provisional diagnosis in need of further study, BED is characterized by recurrent binge eating that occurs in the absence of regular compensatory behaviors (American Psychiatric Association, 1994, 2000). Ample evidence has accumulated that BED is a clinically significant disorder, associated with overweight and obesity, impaired quality of life, and increased general psychopathology and psychiatric comorbidity, especially affective and anxiety disorders (Latner & Clyne, 2008; Striegel-Moore & Franko, 2008; Wilfley, Wilson, & Agras, 2003; Wonderlich, Gordon, Mitchell, Crosby, & Engel, 2009). In anticipation of DSM-V, researchers have begun to empirically examine the boundaries of BED within the eating and weight disorders spectrum, using specific analytical procedures to examine their latent structure, such as taxometric or latent class analysis (Bulik, Sullivan, & Kendler, 2000; Eddy, Crosby, Keel et al., 2009; Mitchell, Crosby, Wonderlich et al., 2007; Striegel-Moore, Franko, Thompson et al., 2005; Wade, Crosby, & Martin, 2006; Williamson, Womble, Smeets et al., 2002). In contrast, the nature of the relationship between BED and other co-occurring psychiatric disorders has received little attention (see Wonderlich, Joiner, Keel, Williamson, & Crosby, 2007).

Psychiatric comorbidity in BED is important to our understanding of this disorder. Some researchers have proposed that BED is a marker of psychopathology within obese individuals rather than a separate mental disorder, due to its fluctuating course and non-specific response to pharmacological and psychological treatment (Stunkard & Allison, 2003). Although the validity of this position has been increasingly challenged by evidence documenting BED’s stability (Fichter, Quadflieg, & Hedlund, 2008; Hudson, Hiripi, Pope, & Kessler, 2007), normative placebo-response (Jacobs-Pilipski, Wilfley, Crow et al., 2007), and specific responsiveness to psychological treatment (Grilo, Masheb, & Wilson, 2006; Masheb & Grilo, 2007; Wilson, Wilfley, Agras, & Bryson, 2010), it remains unknown how strongly BED and major comorbid conditions such as affective and anxiety disorders are related at the latent level. If BED is an associated feature of affective or anxiety disorders, it should be more likely to co-occur with these conditions than to present without them. In addition, the question has been raised as to whether BED is characterized by an underlying affective (Devlin, Goldfein, & Dobrow, 2003) or anxiety disorder. If this was the case, diagnostic indicators of affective or anxiety disorders should at the latent level be more characteristic of BED than its own diagnostic criteria. These are questions that await examination.

In this context, the goal of the present study was to examine the latent structure of BED in relation to that of comorbid psychiatric disorders in order to elucidate (1) whether BED is an associated feature of affective and anxiety disorders and (2) whether it is characterized by an underlying affective or anxiety disorder. For external validation, associations of latent structures were examined with clinically relevant parameters of eating disorder and general psychopathology, health care use, social adaptation, and etiological factors.

## Method

### Design and Recruitment

Under the auspices of the *New England Women’s Health Project,* BED cases, psychiatric controls, and non-psychiatric controls were recruited using two recruitment avenues: The first avenue involved telephone recruitment utilizing a consumer information database of 10,000 women; the second avenue consisted of an advertising campaign using posters, newspaper advertisements, community referrals, and public service announcements.

After completion of a telephone screening interview, eligible individuals were invited to participate in the study that included diagnostic interviews, a risk factor interview, and several self-report instruments. Body weight and height were measured. The institutional review boards at Wesleyan and Columbia Universities approved this study. (For further methodological detail, see Pike, Hilbert, Wilfley et al., 2008; Striegel-Moore et al., 2005).

### Participants

Participants in this study were 151 women with BED as their primary diagnosis, 102 women with non-eating disorder DSM-IV psychiatric diagnoses (PC group), and 259 women with no psychiatric diagnosis (NC group). Exclusion criteria for all groups were physical conditions known to influence eating or weight, current pregnancy, or presence of a psychotic disorder. For the BED group, inclusion required presence of all DSM-IV criteria for BED. Diagnosis of current BED was ascertained through the Eating Disorder Examination (EDE; Fairburn & Cooper, 1993), a semi-structured, investigator-based interview, considered to be the gold standard in eating disorder diagnosis. For the PC group, inclusion required the presence of a lifetime DSM-IV axis I affective or anxiety disorder, but no history of clinically significant eating disorder symptoms. Psychiatric diagnoses other than eating disorders were made using the Structured Clinical Interview for DSM-IV Axis I Disorders (SCID-I; First, Spitzer, Gibbon, & Williams, 1997), a well-established, semi-structured diagnostic interview. For the NC group, inclusion required absence of past or current clinically significant eating disorder symptoms and absence of a current psychiatric disorder. Both diagnostic interviews were conducted by trained assessors (bachelor level or higher) who received ongoing supervision to ensure standardized administration.

Sociodemographic and clinical characteristics are presented in Table 1. The BED group had a higher BMI than the other study groups and was more racially diverse than the PC group (*p* < .001; see Hudson et al., 2007; Striegel-Moore et al., 2005). Both clinical groups had higher rates of lifetime and current affective and anxiety disorders than the NC group, and the PC group had higher rates of lifetime and current anxiety disorders than the BED group (all *p* < .001). A total of N = 140 probands had a current affective or anxiety disorder.

### Assessment

Diagnostic indicators of BED were derived from the Eating Disorder Examination-Questionnaire (EDE-Q; Fairburn & Beglin, 1994), the self-report questionnaire version of the EDE, that was administered to the total sample. The diagnostic indicators of the EDE-Q included frequencies of Objective Bulimic Episodes (OBEs), Subjective Bulimic Episodes (SBEs), and episodes of Compensatory Behaviors over the past 28 days (i.e. total episodes of self-induced vomiting, laxative or diuretic misuse, or excessive exercising), and the degree of Overvaluation of Shape or Weight (composite item, ranging from 0 to 6, with higher scores indicating more severe overvaluation), a criterion that has been recommended as a diagnostic criterion or specifier in the forthcoming revision of the DSM (Latner & Clyne, 2008; Wilfley, Bishop, Wilson, & Agras, 2007; Wonderlich et al., 2009). In previous studies, stability and convergent validity were adequate for OBEs and Overvaluation of Shape or Weight, while validity was more variable for Compensatory Behaviors and SBEs (Hilbert, Tuschen-Caffier, & Ohms, 2004; Mond, Hay, Rodgers, Owen, & Beumont, 2004; Reas, Grilo, & Masheb, 2006). In the current study, convergent validity of the EDE-Q-derived diagnostic indicators was determined through concordance with the EDE that had been used for ascertaining BED diagnosis in the eating disorder group only. Effect sizes were moderate for the number of OBEs (Kendall’s τ_b_ = .36, *p* < .001) and large for Overvaluation of Shape or Weight (Pearson’s *r* = .61, *p* < .001; evaluation according to Cohen, 1988).

Diagnostic indicators of current mood and anxiety disorders were derived from the SCID-I (First et al., 1997). In order to form quantitative scales, leading diagnostic items were summed after being coded as present or absent for all main affective and anxiety disorders or syndromes (i.e., initial codes 3 = *threshold* were recoded as 1, while initial codes 1 = *absent* and 2 = *subthreshold* were recoded as 0). For example, for current Major Depressive Episode, the SCID-I items of depressed mood (A1), lack of interest (A2), change of weight (A3), insomnia/hypersomnia (A6), psychomotor agitation/retardation (A9), lack of energy (A12), feelings of worthlessness (A13), poor concentration (A16), and suicidal tendencies (A19) were summed, creating a scale ranging from 0 to 9. Likewise, leading symptoms for other main mood and anxiety disorders or syndromes were summed: Manic Episode (8 items), Dysthymic Episode (7 Items), Panic Attack (4 items), Agoraphobia (2 items), Social Phobia (4 items), Specific Phobia (4 items), Obessive-Compulsiveness (6 items), Posttraumatic Stress (5 items), and Generalized Anxiety (3 items). (A detailed list of items is available upon request).

### Validators

As a measure of eating disorder psychopathology, the EDE-Q subscale composites of Restraint and Eating Concern were used (5 items each, ranging from 0 to 6, with higher scores indicating more psychopathology). Both subscales have demonstrated adequate internal consistency, stability, and convergent validity (Peterson et al., 2007; Reas et al., 2006). For the current study sample, internal consistencies as measured by Cronbach a were .78 for Restraint and .86 for Eating Concern.

General psychopathology was assessed through the index of global severity (GSI) from the Brief Symptom Inventory (BSI; Derogatis, 1977). Scores range from 0 to 100, with higher scores indicating more severe psychiatric symptoms, and were T-standardized. The GSI-BSI has good internal consistency and is highly correlated with the GSI of the lengthier, well-established Symptom-Checklist-90-R. Internal consistency of the GSI in the current study sample was Cronbach α = .97.

Health Services Use was determined by whether participants reported any medical or psychotherapeutic consultations, emergency room visits, or partial or full hospitalizations in the year prior to assessment (total score ranging from 0 to 5; Striegel-Moore, Dohm, Wilfley et al., 2004).

The Social Adjustment Scale (SAS; Weissman & Bothwell, 1976) was used as a general measure of social functioning in a broad range of domains (e.g., role performance, interpersonal relationships, social and leisure activities). Scores range from 0 to 5, with higher scores indicating poorer social functioning. The SAS has good reliability and validity (Goldman, Skodol, & Lave, 1992; Weissman, Prusoff, Thompson, Harding, & Myers, 1978), and in the current study, mean Cronbach α for the SAS subscales was .78.

Early life experiences were assessed retrospectively using the Oxford Risk Factor Interview (RFI; Fairburn, Doll, Welch et al., 1998). The RFI assesses biological, psychological, and social factors believed to increase risk for the development of an eating disorder. Assessment focuses on the period before the “index age,” that is, the age at which significant and persistent eating disorder symptoms first appeared. Individuals in the PC and in the NC group were assigned the index age of the BED case for which they had served as a comparison subject. Scores of the risk factor items range from 0 to 4, with higher scores indicating more severe or frequent exposure. In order to reduce the likelihood of false positives, data were recoded into 1 = *definite exposure* (initially coded 3 or 4) versus 0 = *no definite exposure* (initially coded 0, 1, or 2). Based on factor analytic procedures (see Striegel-Moore et al., 2005), seven composite risk factor domains were derived from the individual risk factor items and considered in the current report: Participant’s Mental Health, Participant’s Physical Health, Other Environmental Experiences, Family Weight and Eating Concerns, Quality of Parenting, Parental Psychopathology, and Childhood Abuse.

### Data Analysis

The taxometric method is a family of statistical procedures designed to determine latent structures of phenomena and is well-suited for research on the classification of disorders in relation to co-occurring conditions (for methodological detail see Beauchaine, 2007; Ruscio et al., 2004, 2006). Taxometric procedures explore the associations among manifest indicator variables to determine whether the underlying structure is dimensional and consists of a single latent continuum, or taxonic and consists of two latent classes (i.e. a taxon and its complement).

#### Indicator selection

The taxometric method requires that variables used as indicators of the target construct distinguish the putative taxon and its complement with sufficient validity (usually *d* ≥ 1.25) and be substantially less correlated within these classes than in the full sample (nuisance covariance; Meehl, 1995; Ruscio et al., 2006). To address these requirements, validity was estimated by examining the separation of the putative taxa and complements on each indicator variable, and by examining the correlation between them within the putative taxa and complements in a preparatory step. All variables were *z-*standardized prior to analyses.

#### Taxometric analyses

The taxometric analyses pursued the following steps: In order to determine (1) whether BED is an associated feature of affective and anxiety disorders, the taxonicity or continuity of BED was established using the diagnostic indicators for BED; the taxonicity or continuity of current affective and anxiety disorders was determined using the diagnostic indicators of affective and anxiety disorders; and the association between the latent structures of the identified taxa or continua of BED and affective/anxiety disorders was examined (Ruscio & Ruscio, 2004). For each individual, the taxon membership or location on a continuum was estimated using the base-rate technique (Ruscio, 2009). Individuals’ estimated class membership or dimensional scores were then used to estimate the degree of association between the two latent constructs as a test of diagnostic co-occurrence. In order to address (2) whether there is an underlying affective or anxiety disorder in BED, the strength of association of diagnostic indicators of both BED and affective and anxiety disorders with membership to the BED taxon was examined, clarifying whether the diagnostic indicators of BED were more characteristic of that latent structure than the diagnostic indicators of affective and anxiety disorders.

Data were submitted to the taxometric procedure MAMBAC (mean above minus below a cut; Meehl & Yonce, 1994), programmed in R. The *SD* of the base rate estimate was evaluated. For bootstrapping, the averaged empirical curves were compared to those derived on the basis of simulated taxonic and dimensional comparison data (Ruscio & Marcus, 2007). The curves yielded by these procedures were then inspected for evidence of taxonic or dimensional structure by *n* = 24 independent raters, blind to study design and hypotheses (−1 = *taxonic*, 0 = *neither/nor*, 1 = *dimensional*). A comparison curve fit index (CCFI) was calculated to quantify the match between the results for research data and those of taxonic and dimensional comparison data. CCFI values can range from 0 = *dimensional* to 1 = *taxonic*, with values < .40 indicating a dimensional structure, and values > .60 indicating a taxonic structure.

#### Consistency check

As a consistency check, the taxometric procedure MAXSLOPE (maximum slope) (Grove, 2004, 2005), programmed in R, and latent profile analysis (LPA; Latent Gold 4.5) were conducted using the same quantitative indicators as in the MAMBAC analyses. For MAXSLOPE, the *SD* of the base rate estimate and the goodness of fit index (GFI; Waller & Meehl, 1998) were considered for evaluation of latent structure. The curves yielded by MAXSLOPE were inspected for evidence of taxonic or dimensional structure by *n* = 24 independent raters, blind to study design and hypotheses (−1 = *non-linear increase or decrease* [i.e. taxonic], 0 = *neither/nor*, 1 = *no non-linear increase or decrease* [i.e. dimensional]). In the taxonic case, consistency of individuals’ assignment to latent structures by MAMBAC and by MAXSLOPE was evaluated using the tetrachoric correlation coefficient.

In addition, LPA was used as a consistency check (as recommended by R. Crosby, personal communication, 01.09.2009). LPA models varying the number of LPs from 1 to 4 were evaluated, using parameters estimated by maximum likelihood. The number of clusters was determined through minimization of the sample-size adjusted Bayesian information criteria parsimony index (Sclove, 1987) and of the consistent Akaike information criterion (Bozdogan, 1987). Assignment of cluster membership was based on Bayesian probabilities. In cases in which taxometric analysis revealed a latent taxon/complement, LPA was expected to produce at least two latent profiles. If taxometric analyses were suggestive of a latent dimension, LPA was expected to identify one single latent profile. In order to detect spurious classes (Uebersax, 1999), plots of classes across indicators were visually inspected for parallel lines. In the taxonic case, consistency of individuals’ assignment to latent structures by taxometric analyses and by LPA was evaluated using the tetrachoric correlation coefficient.

#### Analysis of diagnostic co-occurrence

The strength of association between the latent structure of BED and diagnostic indicators of BED, and of affective and anxiety disorders, was examined using biserial correlation coefficients, interpreted according to Cohen (1988; small: *r* ≥ 0.10, medium: *r* ≥ 0.30, large: *r* ≥ 0.50) and compared using *z* tests for correlation coefficients.

#### Validation analyses

For validation analyses, comparisons of health services use, social adaptation, risk factors, eating disorder psychopathology, and general psychopathology by individuals’ estimated class membership or dimensional scores were conducted. These were based on GLM and post hoc Tukey tests for continuous variables, χ^2^ tests for categorical variables, or correlation analyses (product-moment, Kendall’s τ_b_, or point-biserial correlation coefficients; analyses conducted using SPSS 17.0). Significance level for all statistical analyses was set at a two-tailed α < .05 (for post hoc analyses, α < .01).

### Preliminary Analysis: Indicator Selection

#### Latent structure of BED

Within the diagnostic indicators of BED (OBEs, SBEs, Overvaluation of Shape or Weight, Compensatory Behaviors), SBEs and Compensatory Behaviors did not sufficiently discriminate between the putative taxon of BED versus the putative complement of PC and NC (*d* < 1.25). In addition, the nuisance covariation criterion was violated for SBEs that showed similar correlations with Overvaluation of Shape or Weight and Compensatory Behaviors in the full sample as in the putative taxon and/or complement (full sample *r* = .41 vs. taxon *r* = .12, complement *r* = .39; full sample *r* = .31 vs. taxon *r* = .24, complement *r* = .26). Formation of a composite from OBEs and SBEs did not lead to a better nuisance covariation. Therefore, the indicator variables of OBEs and Overvaluation of Shape or Weight were selected for taxometric analysis. Summary statistics for all selected indicators are presented in Table 2.

#### Latent structure of affective and anxiety disorders

The SCID-I items for current affective and anxiety disorders had low validity to discriminate between the putative taxon of current affective and anxiety disorders and the putative complement. Only Major Depressive Episode yielded a *d* ≥ 1.25. As the correlation matrix did not display clear indication to group items, composite diagnostic indicators (i.e. total number of symptoms) of affective disorders and anxiety disorders were formed. These composite indicators displayed sufficient validity (*d* = 1.97, 1.67) and satisfied the nuisance covariance criterion (see Table 2).

## Results

### (1) Is BED an Associated Feature of Affective and Anxiety Disorders?

#### Latent structure of BED

**Figure 1:**
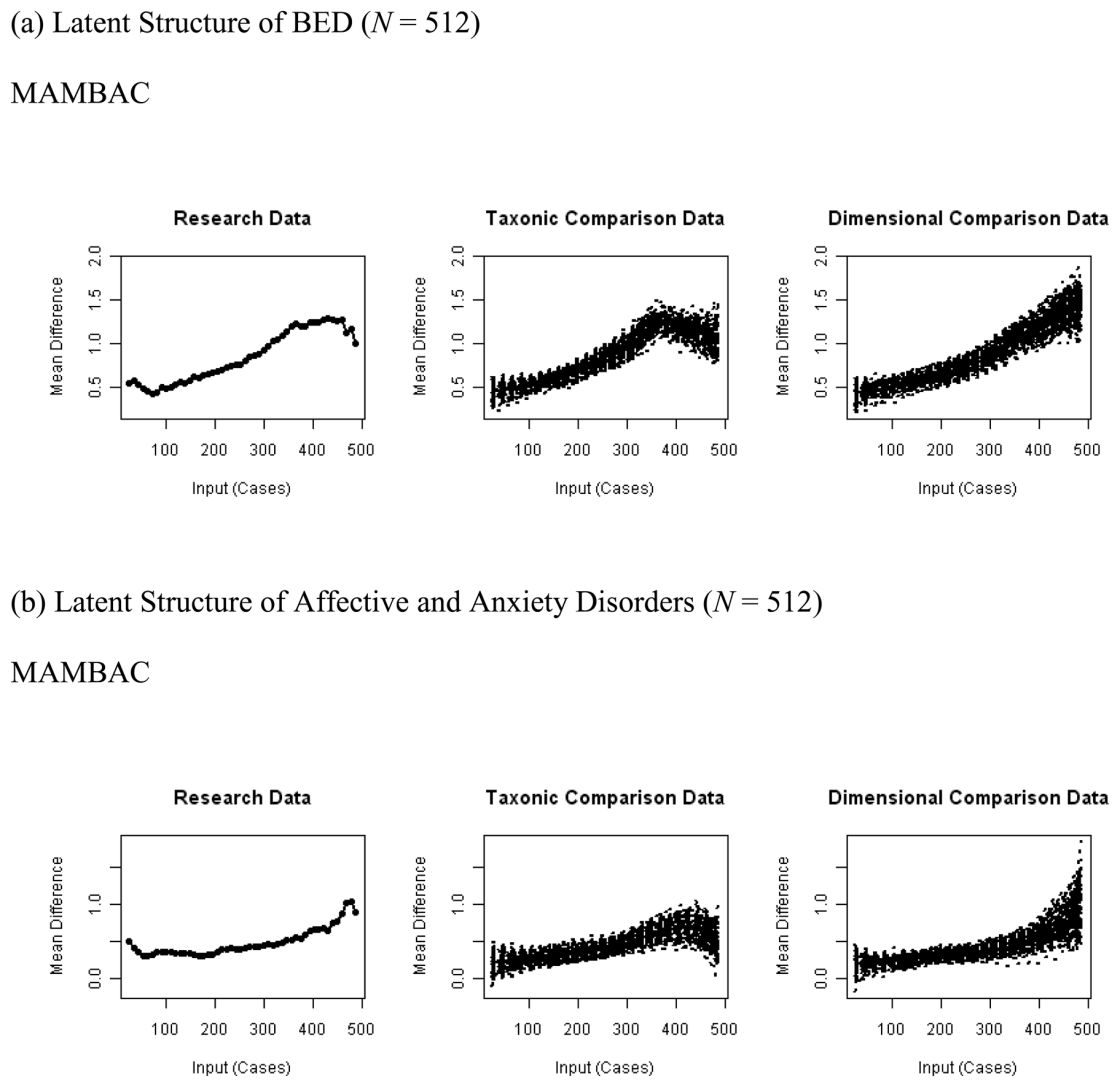
Empirical and Simulated Averaged Curves From Taxometric Analyses. *Note.* MAMBAC indicates mean above minus below a cut.

In the MAMBAC analysis of the latent structure of BED, both curves contained peaked shapes consistent with taxonic structure. The averaged curve, shown in Figure 1, was more similar to that for taxonic than dimensional comparison data; in fact, all independent, blind raters indicated a taxonic structure (24/24, 100.0%). The CCFI > .60 and small *SD* of base rate estimates (Table 3) provided further evidence of taxonic structure.

Consistently, the MAXSLOPE analysis yielded curves with a non-linear increase. All independent, blind raters indicated a taxonic structure (24/24, 100.0%). The large GFI (0.97) and small *SD* of base rate estimates (0.00) provided further evidence of taxonic structure. Consistency with the MAMBAC solution was high (tetrachoric *r_tetr_* = .94, *p* < .001). Further, the LPA produced a 2-profile solution (χ^2^(69, *N* = 512) = 59.29, *p* = .79), demonstrating high consistency with the MAMBAC solution (*r_tetr_* = .98, *p* < .001).

Overall, the evidence was in support of a taxonic structure. The taxon as derived from the MAMBAC analysis included 158 individuals (30.9% out of 512): 124 individuals with BED (78.5% out of 158), 12 individuals from the NC group (7.6%), and 22 individuals from the PC group (13.9%) (χ^2^(2, *N* = 512) = 273.58, *p* < .001). As indicated by large effect sizes, the individuals assigned to the taxon had greater values for OBEs and Overvaluation of Shape or Weight than individuals assigned to the complement (*d* = 1.67, 3.21).

#### Latent structure of affective and anxiety disorders

In the MAMBAC analysis of the latent structure of affective and anxiety disorders, both curves contained peaks, but these peaks appeared at the very right hand of the distribution and were not clearly marked. The averaged curve, depicted in Figure 1, provided some evidence of a taxonic structure, albeit not unambiguously; visual inspection through independent, blind raters yielded in 66.7% (16/24) of raters evidence of a taxonic structure. The low *SD* of base rate estimates provided evidence for a taxonic structure, and the CCFI score was neither clearly in favor nor against a taxonic structure, as CCFI scores between .40 and .60 should be interpreted with caution.

The MAXSLOPE analysis yielded one curve with a non-linear increase (affective disorders, anxiety disorders) and one with a rather linear increase (anxiety disorders, affective disorders): For the first curve, 75.0% of independent, blind raters raters (18/24) indicated a non-linear increase, while for the second curve, 41.7% indicated a non-linear increase (10/24) Overall, the large GFI (1.00) and small *SD* of base rate estimates (0.00) provided evidence of taxonic structure. Consistency with the MAMBAC solution was high (*r_tetr_* = .88, *p* < .001). The LPA produced a 2-profile solution (χ^2^(270, *N* = 512) = 199.00, *p* > .99) that was highly consistent with the taxonic structure identified in the MAMBAC analysis (*r_tetr_* = 1.00, *p* < .001).

Overall, most of the evidence was in support of a taxonic structure for affective and anxiety disorders, but not unambiguously. Further analysis therefore used a taxon, comprised by 92 individuals (18.0% out of 512): 54 individuals with BED (35.8% out of 92), 1 individual from the NC group (0.4%), and 37 individuals from the PC group (36.3%) (χ^2^(2, *N* = 512) = 109.94, *p* < .001). Demonstrated by large effect sizes, the taxon was characterized by greater values for affective and anxiety disorders than the complement (*d* = 1.25, 2.24).

#### Diagnostic co-occurrence

For the analysis of diagnostic co-occurrence, the product of the base rates of both diagnoses at the manifest level (BED diagnosis: 151/512 = 0.29; diagnosis of affective or anxiety disorder: 0.27 = 140/512; product of base rates: 0.29 × 0.27 = 0.08) was compared with the co-occurrence of the taxa of BED and of affective and anxiety disorders at the latent level (55/512 = 0.11; see Table 4), following the analytic approach described by Ruscio and Ruscio (2004). The co-occurrence at the latent level exceeded that of the manifest level (0.11 > 0.08, *z* test, *p* = .017), suggesting that the two conditions co-occurred at greater-than-chance level. The latent taxa were statistically independent (χ^2^(1, *N* = 512) = 43.97, *p* < .001). The BED taxon was approximately twice as likely to occur without the taxon of affective or anxiety disorders (0.65, 103/158) than to co-occur with this taxon (0.35, 55/158) (χ^2^(1, *N* = 158) = 14.58, *p* < .01).

#### Validation analyses

As shown in Table 5, individuals in the BED taxon with and without co-occurring affective or anxiety disorders had similar eating disorder psychopathology, health services use, and early childhood experiences (*p* > .01). Diagnostic co-occurrence within the BED taxon indicated greater general psychopathology, lower social adaptation, and greater premorbid exposure to parental mood and substance disorder (*p* < .01). Individuals from the BED taxon with and without co-occurring affective or anxiety disorders were significantly different from individuals from the affective or anxiety disorders taxon without co-occurring BED on eating disorder psychopathology (*p* < .01), but not on general psychopathology, health services use, social adaptation, or early life experiences (*p* > .01). Both taxa with and without diagnostic co-occurrence differed significantly on nearly all validators from the complement (*p* < .01). There were no group differences on sociodemographic characteristics including BMI (*p* > .05).

### (2) Is BED Characterized by an Underlying Affective Disorder or Anxiety Disorder?

When examining the strength of association between the membership to the BED taxon and single diagnostic indicators, large biserial correlations for OBEs and Overvaluation of Shape or Weight (*r_bis_* = .88, 1.00) and medium correlations for affective disorder and anxiety disorder (*r_bis_* = .36, = .33; all *p* < .001) were revealed. The strength of association between the diagnostic indicators of BED and membership of the BED taxon was significantly greater than that between the diagnostic indicators of affective and anxiety disorders and membership of the BED taxon (*z* tests of correlation coefficients; all *p* < .001).

## Discussion

Comorbid psychopathology continues to complicate the assessment, diagnosis, and treatment of eating disorders, including BED. This raises the question as to whether and how shared characteristics of mental disorders should be considered in classification. An important criterion for recognizing a clinical syndrome as appropriate for inclusion in the DSM as a distinct disorder is whether it can be distinguished from other mental disorders already represented in the DSM (Blashfield, Sprock, & Fuller, 1990).

### (1) Is BED an Associated Feature of Affective and Anxiety Disorders?

The current study elaborated on the boundaries between BED and other mental disorders that are frequently comorbid with BED. Previous studies examining the boundaries between BED and other eating disorders or normality provided initial support for a latent entity of BED (e.g., Bulik et al., 2000; Eddy et al., 2009; Mitchell et al., 2007; Striegel-Moore et al., 2005; Wade et al., 2006; Williamson et al., 2002), but this support was not consistent (Hay, Fairburn, & Doll, 1996). Our latent structure analysis showed that at the latent level BED was co-occurring with, yet distinct from, affective and anxiety disorders. Diagnostic co-occurrence was demonstrated at an above-chance level, suggesting that individuals with both BED and affective disorders have shared characteristics that may be maintained by the association between negative mood or anxiety, and binge eating behavior (Binford, Pederson Mussell, Peterson, Crow, & Mitchell, 2004; Hilbert & Tuschen-Caffier, 2007; Stein et al., 2007) or weight and shape concern (Hilbert, Tuschen-Caffier, & Vögele, 2002; Masheb & Grilo, 2003; Svaldi, Caffier, Blechert, & Tuschen-Caffier, 2009). Shared characteristics could stem from exposure to similar risk factors such as impairments in mental or physical health, abusive and disruptive experiences, and familial problems, as suggested by retrospective risk correlates (see also Fairburn et al., 1998). Alternatively, comorbid mental disorders could also represent a consequence of the eating disorder (Mussell, Mitchell, Weller et al., 1995). Longitudinal research is needed to examine the association of BED and co-occurring conditions over time.

Despite diagnostic co-occurrence of BED and affective and anxiety disorders, the probability for BED to occur *without* an affective or anxiety disorder was twice as large as to co-occur *with* these conditions at the latent level. Diagnostic co-occurrence within the BED taxon indicated greater general psychopathology, lower social adaptation, and greater premorbid exposure to parental mood and substance use disorder, but not greater eating disorder psychopathology. Previous research has at the manifest level consistently documented associations between psychiatric comorbidity and general psychopathology in clinical samples of BED (Grilo et al., 2009; Peterson, Miller, Crow, Thuras, & Mitchell, 2005). In contrast to the current and another clinical study’s results (Wilfley et al., 2000), these studies also found significantly greater eating disorder psychopathology in patients with BED with psychiatric comorbidity than in patients with BED without psychiatric comorbidity. Methodological differences in sampling, operationalization, and level of analysis may account for these inconsistencies. However, it is also noted that the differences in eating disorder psychopathology by psychiatric comorbidity that have been found in previous studies were mostly small.

Eating disorder psychopathology discriminated individuals in the BED taxon from individuals in the affective and anxiety disorders taxon. These results from the current study suggest that the specific eating disorder psychopathology can be captured with eating-disorder-specific diagnostic indicators, but would largely not be captured when using only indicators of affective or anxiety disorders. In addition, this study found that women in the BED taxon with or without co-occurrence differed from the complement in nearly all validators. The latent taxon of BED was composed of more than two-thirds of individuals with a manifest diagnosis of BED; thus, this diagnostic entity was mostly reproduced, although for this taxometric analysis only a subset of self-reported diagnostic indicators was available. Delineation of the boundary between BED and obesity was not the focus of the current study; the non-eating disordered control sample was not matched to the BED sample on BMI. Nevertheless, these results underscore that BED is not an associated feature of comorbid psychopathology but should be conceptualized as an eating disorder with substantial comorbid psychopathology (see Devlin et al., 2003).

### (2) Is BED Characterized by an Underlying Affective Disorder or Anxiety Disorder?

The diagnostic indicators of BED were more characteristic of the latent taxon of BED than indicators of affective or anxiety disorders, suggesting that BED is not an associated feature of an underlying affective or anxiety disorder. It is noteworthy that these results refer to BED as a whole. Based on the evidence from suptyping by negative mood at the manifest level (Chen, McCloskey, & Keenan, 2009; Jansen, Havermans, Nederkoorn, & Roefs, 2008; Masheb & Grilo, 2008; Stice, Davis, Miller, & Marti, 2008) it is possible that for a subset of individuals with BED (e.g., those from the high negative affect subtype), the indicators of affective or anxiety disorders or of a specific affective or anxiety disorder are more characteristic than the indicators of BED. Future research can address these questions in latent structure analyses with adequate sampling of individuals from the high versus low negative affect subtype and of specific major affective and anxiety disorders. Although the results are overall not suggestive of an underlying affective or anxiety disorder in BED, the medium-size associations between indicators of affective and anxiety disorders and membership in the BED taxon suggest, in accordance with previous research, that general psychopathology is a characteristic of this disorder for a subset of individuals.

#### Methodological Considerations

Our findings need to be considered in light of strengths and limitations of the current study. We utilized well-characterized community samples of women with BED, affective and anxiety disorders, and no current psychiatric diagnosis. Although an unselected sample would have been preferable for these analyses, with a low base rate entity such as BED more than 20,000 individuals would have been required to detect a BED taxon. In this case, oversampling of BED is permissible (Ruscio & Ruscio, 2004). However, as the exclusion of eating disorders from both control groups may have increased separation between groups, replication of results in an unselected sample is warranted. The measures used had established reliability and validity. Standardized administration of diagnostic interviews was ensured through training and supervision, but information on interrater reliability was not available. For the latent structure analysis, diagnoses were based on diagnostic items from clinical interview or self-report-questionnaire that had overlap but were not fully congruent with criteria used to form the diagnostic groups. For example, the BED diagnostic indicators used in the taxometric analysis based on self-report did not include behavioral indicators of loss of control or of distress over binge eating; both criteria have been critized for lacking empirical support (Latner & Clyne, 2007). This contributed to a low number of indicators available for the current analysis, and as a consequence, MAMBAC results could only be corroborated with the taxometric method MAXSLOPE that also requires only two indicators, but is less commonly used in latent structure analyses than other procedures. We used LPA analysis to check consistency and overcome this limitation. A further limitation is that the evidence was mostly – but not unambiguously – in support of a taxonic structure of affective and anxiety disorders, which is nevertheless consistent with the literature. Taxometric analyses of major depression have provided evidence for a taxonic structure when assessment of depression was based on interview, but not when based on self-report (Ruscio, Brown, & Ruscio, 2009; Ruscio & Marcus, 2007). For anxiety disorders, there is some evidence of a taxonic structure of severe anxiety symptoms (Kotov, Schmidt, Lerew, Joiner, & Ialongo, 2005) and mixed anxiety and depression in youth (Schmidt et al., 2009), whereas evidence on agoraphobia, social phobic fears, worry, posttraumatic stress, or other fears are mostly suggestive of a dimensional structure (Broman-Fulks, Ruggiero, Green et al., 2006; Forbes, Haslam, Williams, & Creamer, 2005; Haslam, 2003; Kollman, Brown, Liverant, & Hofmann, 2006; Ruscio, Borkovec, & Ruscio, 2001; Slade & Grisham, 2009; Weeks, Norton, & Heimberg, 2009). Collapsing affective and anxiety disorders into one category is supported by structural evidence indicating that affective and anxiety disorders fall within one internalizing spectrum, although there may be depression- and anxiety-related subclasses among them (Watson, 2005). Finally, we were able to use for validation analyses clinically informative correlates such as health services utilization and social adaptation as well as data about early life experiences in order to gain insight into retrospectively gathered etiological patterns.

#### Implications

The results of the current study have important implications for the understanding of the nature of BED. By demonstrating distinctiveness of BED from affective and anxiety disorders at the latent level, the results contribute to the construct validity of this disorder. Future research will need to determine BED’s association with single affective or anxiety disorders and other comorbid conditions such as substance use disorder (Lilenfeld, Ringham, Kalarchian, & Marcus, 2008), personality disorders (Wonderlich et al., 2007), and obesity (Devlin, Goldfein, Petkova, Liu, & Walsh, 2007; Wonderlich et al., 2009). Our results further show that OBEs and overvaluation of shape or weight are central for the construct and classification of BED (Grilo, Hrabosky, White et al., 2008; Grilo et al., 2009; Hrabosky, Masheb, White, & Grilo, 2007; Mond, Hay, Rodgers, & Owen, 2007; Goldschmidt et al., 2010; Grilo, Masheb, & White, 2010), while characteristics of affective and anxiety disorders do not seem suited to fully capture the specific psychopathology of BED.

Clinically, similar to previous findings on subtyping by current or lifetime depressive disorders (Grilo, Masheb, & Wilson, 2001; Peterson et al., 2005), the results advocate for a thorough assessment of comorbid psychiatric disorders as an indicator of greater general psychopathology and less social adaptation, as psychiatric comorbidity predicts a less favorable long-term outcome (Fichter et al., 2008). Psychiatric comorbidity can be used as an indicator of a need for enhanced treatment (Wilson et al., 2010). Further, because the data suggest shared maintenance factors among BED and affective and anxiety disorders, maintenance models should more explicitly take these maintenance factors into account that apply to some, but not all, patients with BED (see Fairburn, Cooper, & Shafran, 2003). Such models could guide treatment design to integrate standard cognitive-behavioral interventions for BED with those for co-occurring pathology, such as self-monitoring of mood- or anxiety-related symptoms, exposure and response prevention for anxiety, cognitive interventions for negative cognitive schemata related to the affective or anxiety disorder, or emotion regulation interventions for irritable mood. Future research may continue to establish (Fairburn, Cooper, Doll et al., 2009) whether treatment approaches that address co-occurring pathology beyond eating disorder psychopathology can lead to an improved treatment outcome.

## Figures and Tables

**Table 1:** Sociodemographic and Clinical Characteristics.

	BED		PC		NC		Univariate tests			Post-hoc tests (*p* < .01)
	*N* = 151		*N* = 102		*N* = 259					

	*M*	*SD*	*M*	*SD*	*M*	*SD*	*F*	*df*	*p*	
Age, Years	31.12	5.81	29.61	6.85	30.07	5.44	2.40	2, 509	.092	
Body Mass Index, kg/m^2^	34.45	9.54	26.04	6.94	25.50	6.23	73.59	2, 509	< .001	BED > PC, NC
	*n*	*%*	*n*	*%*	*n*	*%*	χ^2^	*df*	*p*	
Race							9.91	2	.007	BED > PC
Black	56	37.1	19	18.6	78	30.1				
White	95	62.9	83	81.4	181	69.9				
Education							1.97	4	.741	
High School or Less	30	19.9	21	20.6	41	15.8				
Some College	76	50.3	48	47.1	131	50.6				
College Grad or Higher	45	29.8	33	32.4	87	33.6				
Lifetime Psychiatric Comorbidity										
Affective Disorder	102	67.5	69	67.6	12	4.6	220.86	2	< .001	BED, PC > NC
Anxiety Disorder	61	40.4	69	67.6	14	5.4	79.49	2	< .001	PC > BED > NC
Affective or Anxiety Disorder	119	78.8	102	100.0	24	9.3	323.67	2	< .001	PC > BED > NC
Current Psychiatric Comorbidity										
Affective Disorder	38	25.2	29	28.4	0	0.0	156.20	2	< .001	BED, PC > NC
Anxiety Disorder	38	25.2	57	55.9	0	0.0	157.42	2	< .001	PC > BED > NC
Affective or Anxiety Disorder	62	41.1	78	76.5	0	0.0	235.68	2	< .001	PC > BED > NC

*Note.* BED indicates binge eating disorder; PC, psychiatric control group; NC, non-psychiatric control group.

**Table 2:** Correlations and Validity Estimates for Indicators in Taxometric Analyses (*N* = 512).

(a) Criteria of BED in a BED Taxon (*n* = 151) and Complement (*n* = 361)		
	OBEs	Overvaluation of Shape or Weight
	**Correlations**	
Overvaluation Shape or Weight	.53 (.22/.33)	
	**Validity Estimates**	
Taxon *M* (*SD*)	0.91 (1.17)	−0.53 (0.12)
Complement *M* (*SD*)	0.89 (0.91)	−0.52 (0.60)
Validity (*d*)	2.23	2.01
Skew	2.65	0.58

**(b) Criteria of Affective and Anxiety Disorders in a Taxon of Current Affective and Anxiety Disorders (*n* = 140) and Complement (*n* = 372)**		
	**Correlations**	
	Affective Disorder	Anxiety Disorder
Anxiety Disorder	.28 (.13/−.12)	
	**Validity Estimates**	
Taxon *M* (*SD*)	1.56 (1.63)	1.31 (1.34)
Complement *M* (*SD*)	−0.23 (0.45)	−0.23 (0.71)
Validity (*d*)	1.97	1.67
Skew	2.79	1.97

*Note.* OBEs indicate objective bulimic episodes. Correlation coefficients are displayed as full sample (taxon/complement).

**Table 3:** Estimated Base Rate and Comparison Curve Fit Index (CCFI) for Taxometric Analyses (*N* = 512).

	Taxon Base Rate Estimate	CCFI
	*M* (*SD*)	
Latent Structure of BED		
MAMBAC	.36 (.05)	.62
Latent Structure of Affective and Anxiety Disorders		
MAMBAC	.36 (.01)	.41

*Note.* CCFI ranges from 0 = *dimensional* to 1 = *taxonic*. CCFI > .50 is interpreted as evidence in support of taxonic structure. MAMBAC indicates mean above minus below a cut.

**Table 4:** Co-Occurrence of the Latent Taxa of BED and Affective/Anxiety Disorders (*N* = 512).

		BED Taxon	
		Absent	Present
Affective/Anxiety	Absent	317 (61.9%)	103 (20.1%)
Disorder Taxon	Present	37 (7.2%)	55 (10.7%)

**Table 5:** Validation of Taxa of BED and of Affective and Anxiety Disorders With or Without Diagnostic Co-occurrence (*N* = 512).

	BED Taxon without Co-occurrence		Affective and Anxiety Disorders Taxon without Co-occurrence		Co-occurrence		Complement				
	*N* = 103		*N* = 37		*N* = 55		*N* = 317				

	*M*	*SD*	*M*	*SD*	*M*	*SD*	*M*	*SD*	*F*	*df*	*p*
Psychopathology											
Restraint (EDE-Q)	2.34^a^	1.39	1.01^b^	1.21	2.17^a^	1.36	0.90^b^	1.10	47.46	3, 508	<.001
Eating Concern (EDE-Q)	2.60^a^	1.36	0.65^b^	0.94	2.86^a^	1.03	0.45^b^	0.72	206.93	3, 507	<.001
General Psychopathology (BSI GSI)	66.44^a^	20.89	72.48^a^^b^	26.27	78.26^b^	21.28	49.73^c^	11.63	70.64	3, 492	<.001
Health Services Use	1.61^a^	0.92	1.86^a^	0.83	1.90^a^	1.00	1.30^b^	0.72	13.58	3, 481	<.001
Social Adaptation (SAS)	1.99^a^	0.48	1.93^a^^b^	0.39	2.18^b^	0.52	1.55^c^	0.35	50.87	3, 420	<.001
Early Childhood Experiences (RFI)											
Participant’s Mental Health	0.33^a^	1.38	0.64^a^	1.64	0.71^a^	1.36	−0.56^b^	0.67	45.36	3, 508	<.001
Participant’s Physical Health	0.43^a^	1.96	−0.26^a^^b^	1.58	0.69^a^	2.35	−0.27^a^	1.18	9.74	3, 508	<.001
Sexual and Physical Abuse	0.48^a^	2.48	0.63^a^	2.16	1.26^a^	2.61	−0.56^b^	1.86	16.41	3, 508	<.001
Other Environmental Experiences	0.19^a^	1.61	−0.17^a^^b^	1.26	0.75^a^	1.78	−0.33^b^	1.40	10.00	3, 508	<.001
Family Weight and Eating Concerns	0.14^a^	0.65	−0.31^a^^b^	0.61	0.00^a^	0.81	−0.44^b^	−0.25	17.02	3, 508	<.001
Quality of Parenting	0.49^a^	1.13	0.56^a^	0.99	0.87^a^	1.34	−0.56^b^	1.05	47.81	3, 508	<.001
Parental Mood and Substance Disorder	0.28^a^	2.14	0.67^a^^b^	2.13	1.19^b^	2.57	−0.47^b^	1.64	15.79	3, 508	<.001

*Note.* EDE-Q, Eating Disorder Examination-Questionnaire (range: 0–6*; scores indicating less favorable conditions are asterisked); BSI, Brief Symptom Inventory, GSI, Global Severity Index (T scores); SAS, Social Adjustment Scale (1–5*); RFI, Oxford Risk Factor Interview (average of standardized risk factor scores by risk factor domain; they can be interpreted as deviations from the mean, with higher scores indicating greater exposure).

a,b,c Different superscripts indicate significant post hoc Tukey tests (p < .01) following significant univariate GLM analyses (p < .05).
